# Task Specific and General Patterns of Joint Motion Variability in Upright- and Hand-Standing Postures

**DOI:** 10.3390/e24070909

**Published:** 2022-06-30

**Authors:** Moira Pryhoda, Karl M. Newell, Cassie Wilson, Gareth Irwin

**Affiliations:** 1 Department of Mechanical and Materials Engineering, University of Denver, Denver, CO 80208, USA; moira.pryhoda@du.edu; 2 Department of Kinesiology, University of Georgia, Athens, GA 30602, USA; kmn1@uga.edu; 3 Department for Health, University of Bath, Bath BA2 7AY, UK; c.wilson3@bath.ac.uk; 4 Cardiff School of Sport and Health Science, Cardiff Metropolitan University, Cardiff CF23 6XD, UK

**Keywords:** motor control, postural control, kinematics, gymnastics, center of mass

## Abstract

The preservation of static balance in both upright- and hand-stance is maintained by the projection of center of mass (CM) motion within the region of stability at the respective base of support. This study investigated, from a degrees of freedom (DF) perspective, whether the stability of the CM in both upright- and hand-stances was predicted by the respective dispersion and time-dependent regularity of joint (upright stance—ankle, knee, hip, shoulder, neck; hand stance—wrist, elbow, shoulder, neck) angle and position. Full body three-dimensional (3D) kinematic data were collected on 10 advanced level junior female gymnasts during 30 s floor upright- and hand-stands. For both stances the amount of the dispersion of joint angle and sway motion was higher than that of the CM and center of pressure (CP) with an inverse relation to time-dependent irregularity (SampEn). In upright-standing the variability of neck motion in the anterior–posterior direction was significantly greater than that of most joints consistent with the role of vision in the control of quiet upright posture. The findings support the proposition that there are both task specific and general properties to the global CM control strategy in the balance of upright- and hand-standing induced by the different active skeletal-muscular organization and the degeneracy revealed in the multiple distributional variability patterns of the joint angle and position in 3D.

## 1. Introduction

Humans develop balance control strategies in postural stances at an early age by organizing muscle contractions to create torques about joints to preserve the projection of the center of mass (CM) within the stability region at the base of support [1,2,3]. The experimental evidence for this relation has primarily been from the analysis of quiet upright stance where postural variability has been assessed by the motion of the center of pressure (CP) and/or less frequently that of CM, typically using a single force platform with or without motion capture equipment. The long-standing view of the quiet upright stance has been that it reflects the organization of an inverted pendulum [3,4].

Postural perturbation experiments have shown, however, that ankle, hip, ankle–hip and ankle, knee, and hip strategies can be marshalled rapidly in a reactive mode to preserve the stability of balance [5,6]. Moreover, evidence points to postural stance as a multi-link control system [6,7,8,9,10] though the nature of this multi-link control remains an open challenge. Here we report a detailed analysis of the variability (dispersion and time dependent regularity) of the local component joint degrees of freedom (DF; angle and 3D position) to that of the more global postural variables of CP and CM (that is variables that emerge from a compression of the individual joint motion variability). Of particular interest was the deterministic and stochastic postural variability patterns across joints and tasks and whether they reveal different structures of variability in the time domain that would suggest their different roles in postural control (e.g., [11,12]).

The handstand is a fundamental skill in gymnastics due to its embedding within more complex gymnastic skills and the fact that it is performed on all apparatus in both male and female gymnastics [13]. The restricted stability region of the handstand leads it to require a balance control strategy that is more constrained than that for upright stance [14,15,16]. The motion at four joints (wrist, elbow, shoulders, and hip) has primarily been examined in handstand postural control. Previous work has focused on the sagittal plane, in which torque about the wrist has been found to be the most prevalent control strategy [5] in what was called a ‘strong’ handstand. A strong handstand balances in a straight body line with minimal joint displacement, while weaker handstands may require compensatory torques, utilizing either a shoulder strategy or a hip strategy [17].

The study reported here examined the variability of the upright- and hand-stance postures of advanced level female gymnasts in the context of the collective postural coordination pattern [8,18]. The postural control of both quiet upright- and hand-standing from a systems DF perspective [19] was analyzed through a decomposition of the patterns of the amount of variability of the respective joint motion motions and how well they predicted CM and CP motion. CM-related variables have been viewed as collective variables in upright-standing posture [20,21,22]. Here we examine the role of individual component joint angle and 3D position variables in predicting the motion variability of CM. It was hypothesized that the variability of CM motion would be posture dependent and relatively lower than that of the individual joint motions in both position and angle variables, respectively.

Postural control of limb segments has typically been analyzed through either joint angle motions of the body as a kinematic chain or joint position in the Euclidean frame of reference, which, in effect, provides indices of body sway [4]. The joint angle motions can be considered in an egocentric frame of reference while the joint positions in Euclidean space are viewed in an allocentric frame of reference [5]. The contrasting influence of these variables to predict CM stability was analyzed in the context of an anatomical or task-oriented organization to the structure of the relative variability at the joints [23,24]. We examined the hypothesis that the pattern of the amount of CM motion variability is driven by the respective task constraint of whether it is the hands or feet in contact with the surface of support, rather than a universal fixed anatomical proximal-distal pattern of joint angle or 3D sway motion [24]. A measure of neck motion variability in the control of the two postures was also included in the analysis [25,26,27]. The role of neck motion in posture is typically not analyzed, except in manipulations of vestibular control, but it links directly to the established finding of a strong role of visual information in postural control (e.g., [28,29,30]).

The variability analyses of posture focused on the amount (dispersion) and time-dependent irregularity of intra-gymnast variability of key variables in execution of the two postural tasks. The analysis of the time-dependent entropy (uncertainty) was assessed through the irregularity/regularity entropy measure of SampEn [31]. Past work in the variability of motor control has shown an inverse relation between the amount and the time-dependent structure of movement variability in a range of postural and movement tasks [32]. That is enhanced irregularity of a time series has been associated with a lower amount of variability (dispersion) of the respective signal and vice versa. We examined whether this relation held across or was unique to the different categories of variables (joint angle, joint 3D position, in contrast to CM, CP) in multi-joint quiet upright- and hand-standing postural tasks.

## 2. Materials and Methods

### 2.1. Participants

Ten competitive team gymnasts, enrolled in the USA Gymnastics Development program from gymnastics clubs surrounding Denver, CO, USA, between the ages of 7–13 yrs (11 ± 1.9 yrs), participated in this investigation. Inclusion criteria required the ability to hold a handstand for 30 s on both the floor and the balance beam. Gymnasts who had sustained an upper extremity injury within the past 6 months or who currently had an injury requiring a cast on any limb were not eligible for the study.

### 2.2. Apparatus

Before performing static balance postures, gymnasts were outfitted with 54 reflective markers, including a full lower and upper extremity marker set and an abbreviated head and trunk marker set. An eleven-camera passive motion capture system (Vicon Motion Systems) was used to capture full body segment motion at 100 Hz using Vicon Nexus Capture software (Version 2.9, Motion Systems, Ltd., Oxford, UK). Ground reaction forces were sampled at 1000 Hz from two force platforms (Bertec, Columbus, OH, USA) embedded side-by-side in the laboratory flooring.

### 2.3. Procedures

The gymnasts participated in both an upright-standing balance posture and a static hand-stand balance posture each performed for 30 s. In the upright-standing balance posture, the gymnast maintained a gaze straight forward, hands on hips, and feet shoulder width apart with each foot placed on a separate force platform. In the static handstanding balance posture, the gymnast maintained a gaze on the floor and hands shoulder width apart with each hand placed on a separate force platform. Up to three attempts were given to obtain a 30 s handstand, defined from the point at which the minimum sagittal distance between feet was reached to the point at which the sagittal distance between feet expanded. In the event that a 30 s handstand was not achieved, the longest trial length was used for analysis. The upright- and hand-standing postures reported in this work were part of a larger data collection with 7 total postures, the order of which was randomized.

### 2.4. Data Processing

A biomechanical body model was created in Visual3D (Version v6, C-Motion, Inc., Germantown, MD, USA) for each subject using the motion capture data collected using the full body marker set. Using Dempster’s body segment parameters, the segmental center of mass was calculated as a percent of segment length from the proximal end for each segment. Torque for each segment was then calculated using segment coordinates in the global coordinate system and segmental mass percent. Finally, total torque was used to determine full body CM location; this is a process known as the principle of moments.

Marker and force data were filtered using a 4th order zero-phase-lag Butterworth filter with a 6 Hz cutoff frequency. A sensitivity analysis comparing 6 Hz, 10 Hz, and 20 Hz showed negligible difference and that a 6 Hz filter accommodated the data. For both the upright- and hand-standing postures we calculated: (1) CP position along the medio-lateral (ML) and anterior–posterior (AP) axes; (2) CM position along the ML, AP, and longitudinal (LG) axes; (3) CM-CP inclination angle along the ML and AP axes calculated as the instantaneous angle of the line connecting the CM and CP with respect to vertical [33]; (4) Euclidian joint positions (global coordinate system) of the wrist, elbow, shoulder, hip, knee, ankle, and neck along the ML, AP, and LG axes for the duration of the posture; and (5) 3D joint angles (local coordinate system) were analyzed for the wrist, elbow, shoulder, hip, knee, ankle, and neck. Joint angles were calculated by transforming the distal segment coordinate system to that of the proximal segment using a Cardan sequence of rotations. The neck joint angle was defined by expressing the head coordinate system with respect to the trunk coordinate system [26,27].

The ML, AP, and LG axes for each variable were calculated separately. Joint angles and positions were calculated for the left and right side of the body separately. Coordination analyses in the frequency domain and the coupling of the motion of variables could complement the variability and entropy analyses emphasized here [25] but were considered beyond the scope of this paper.

### 2.5. Statistical Analysis

Within-trial variability is reported as the standard deviation of each variable in each orthogonal-plane of motion (Euclidean joint positions) and about each 3D joint axes (joint angles). Inferential statistics on the many variables was not the focus of this descriptive study on postural variability given that we have a convenience sample of competitive gymnasts.

For each posture, the time series data for all participants were used to run multiple linear regressions, that met assumptions relating to number of events [34]. In the upright-standing posture, the 12 predictor variables included the ankle, knee, hip, and neck, in the three orthogonal axes. In the hand-standing posture, 15 predictor variables included the wrist, elbow, shoulder, hip, and neck in the three orthogonal axes. Regressions for both postures included the neck joint to consider the variability due to visual control. For all regressions, the position or angle time histories of the predictor variables in all three planes of motion were included, creating a total of 12 predictor variables for the upright-standing posture regressions and 15 predictor variables for the hand-standing posture regressions. Joint position predictor variables were regressed against CM position in each of the three planes (sagittal, frontal, and transverse), and joint angle predictor variables were regressed against CM position in all three planes and CM–CP inclination angle in both planes of motion (mediolateral, ML; and anterior–posterior, AP). Potential collinearity was assessed via condition index. Condition indices did not exceed 30, indicating that there were not strong linear relationships between predictor variables [35]. Using standardized coefficients, the predictor variable contribution to the model was converted to a percent.

SampEn was used to assess the regularity/irregularity of each time series [31]. The parameter values were embedding dimension m = 2, tolerance r = 0.2 × SD. The approach to SampEn analysis was parallel to that outlined above for SD. Finally, bivariate regression was performed to examine the correlation between the SD and SampEn across all variables.

## 3. Results

### 3.1. Overall Performance

Each participant held a handstand for an average of 27.36 ± 4.11 s, with six of the ten gymnasts holding the handstand for the full 30 s. The results are organized to allow an examination of the amount of variability (SD) of the two postures (upright- and handstanding), with a focus on the individual Euclidian joint positions, the joint angles, and their collective relation to CM and CP motion. This was followed by a parallel set of analyses of SampEn, including its relation to SD. Failing to hold the handstand for the full 30 s is a form of nonstationarity and we did not analyze this in detail during the trial.

### 3.2. Dispersion (Amount) of Variability

**Figure 1:**
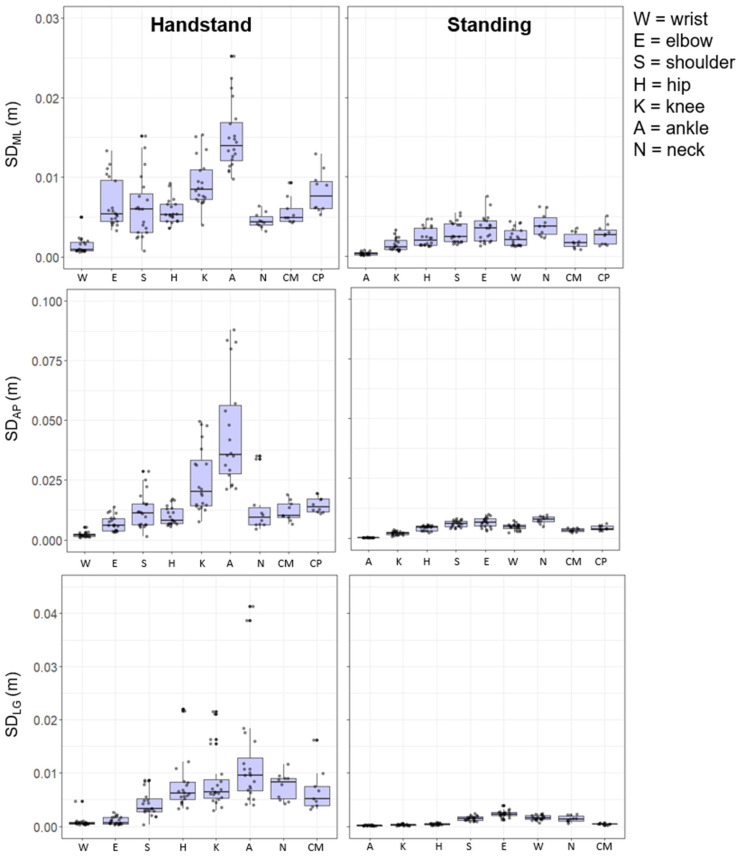
Box plots of the SD of the Euclidian joint positions (m) during hand-stand and upright-standing poses for each gymnast on the right and left side.

**Figure 2:**
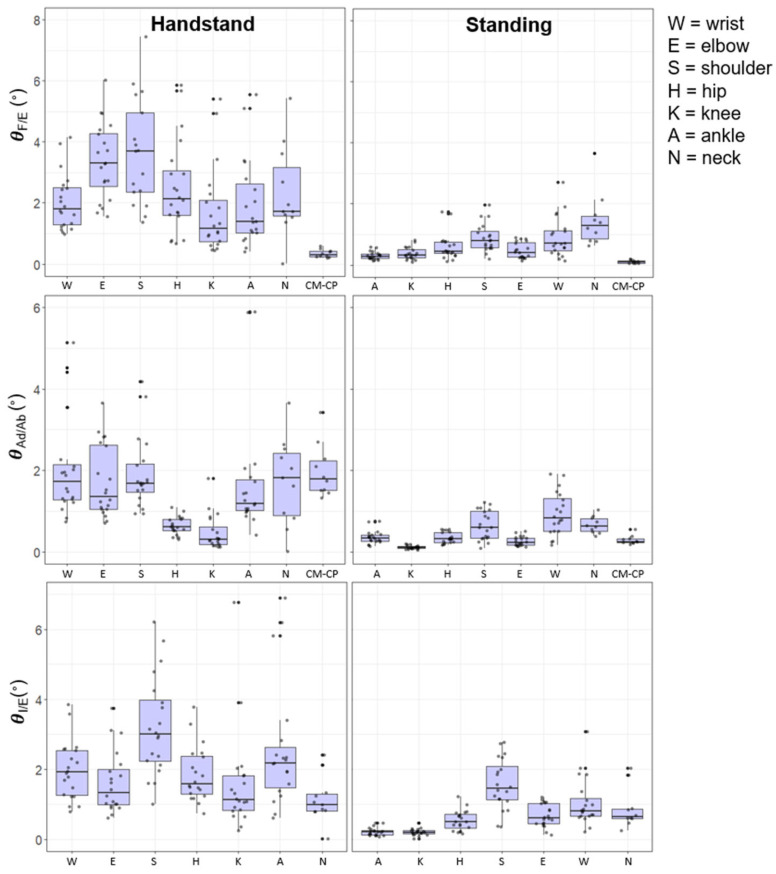
Box plots of the SD of the joint angles (°) during hand-standing and upright-standing poses for each gymnast on the right and left side.

Figure 1 and Figure 2 illustrate the dispersion variability (SD) of the local Euclidian joint positions (m) and joint angles (°) during each posture, respectively. The figures provide details of inter- and intra-gymnast variability, with the former being represented by the size of the box and the latter illustrated by the individual gymnast dots. Overall, the results reveal that variability for both Euclidian joint positions and joint angles was greater, both within and between individuals, for hand-standing compared to upright-standing. The variability of the position of the CM was also higher in hand-standing compared with upright-standing. Specifically in ML, AP, and LG, the hand-standing CM variability was over double that of the respective variables in upright-standing.

### 3.3. Euclidian Joint Position

Figure 1 shows the joint position variability as a function of postural stance. During the hand-standing posture the greatest levels of variability for the Euclidian joint positions were observed at the ankle in the ML (0.012 m), AP (0.035 m), and LG (0.010 m) direction, followed by the knee in the ML (0.008 m) and AP (0.021 m) direction. Conversely, the joint positions with the lowest levels of variability were those closer to the base of support, with wrist joint position (ML = 0.008 m, AP = 0.002 m, LG = 0.005 m) and the elbow (AP = 0.007 m, LG = 0.007 m) (Figure 1).

The upright-standing posture displayed a similar pattern of variability with lowest variability being that of the joint closest to the base of support. As such the ankle and knee joint locations displayed the least amount of variability across all three orthogonal directions (e.g., ankle joint: ML = 0.03 m, AP = 0.02 m, LG = 0.01 m). Variability further away from the base of support was higher with the neck motion demonstrating most variability in the ML and AP directions (0.38 m and 0.74 m) (Figure 1).

It is evident from the joint locations that there are relatively higher levels of variability at the component joint level compared to the global measures of CM and CP for both the hand-standing and upright-standing postures. In addition, in all cases hand-standing showed higher levels of movement variability than upright-standing (Figure 1).

### 3.4. Joint Angles

Figure 2 shows the joint angular variability as a function of posture. The joint angular kinematics showed that during the hand-standing posture the variability was generally greatest at the shoulder joint for extension/flexion (3.11°), adduction/abduction (1.72°), and internal/external rotation (2.39°). The least variable joints were observed to be the knee joint for extension/flexion (1.17°), adduction/abduction (0.32°), and the neck joint (0.87°) for internal/external rotation. In the upright-standing posture the shoulder and the neck joint showed the highest levels of variability in extension/flexion and adduction/abduction although relatively lower in magnitude (Figure 2).

The knee and ankle joint produced the lowest levels of variability across the three orthogonal joint motions (Figure 2). As observed with the global position of the CM and CP the variability of the CM–CP angle showed lower levels of variability than the individual joint angles in hand-standing and upright-standing postures.

### 3.5. Anatomical Sequencing of Variability

During upright-standing the Euclidian joint position variability showed a clear proximal to distal increase in the AP and ML direction. In hand-standing this kinematic sequence showed a contrasting distal to proximal order, in the AP and LG directions, however, there was more variation of the sequence in the ML joint position locations. Variability of flexion/extension showed a clear proximal–distal sequence for upright-standing, but not in hand-standing, although there was an increase in variability from the wrist to elbow joint. The joint motion of abduction/adduction during the upright-standing position showed a more proximal to distal pattern; however, hand-standing showed a similar level of variability across the upper limb joints, i.e., wrist, elbow, and shoulder joints.

Joint position data showed the greatest levels of variability in the AP direction, followed by the MP then LG, for hand-standing and upright-standing posture. The joint angle variability was greatest in extension/flexion followed by abduction/adduction and internal/external rotation (Figure 1 and Figure 2).

### 3.6. Prediction of CM Position

Variables with greater than 10% contribution to the model from the multiple linear regressions with an R^2^ above 0.60 are reported. Table 1 and Table 2 provide the outcome of the multiple regression, highlighting the significant predictor variables for CM motion in all three orthogonal axes for joint positions and joint angles.

Joint position variability of the hip, knee, and neck predicted the CM variability in the AP, ML, and LG directions during the upright-standing posture. During hand-standing the variability of a greater number of joints predicted CM variability (Table 1), specifically, the hip and shoulder in the AP, ML, and LG directions. Contributions to CM variability were also seen from the wrist in CM_AP_ and CM_ML_ and elbow in CM_AP_.

Table 2 shows the correlations between joint angle variability with the variability of the CM. During upright-standing the angles at the ankle and hip contributed to the greatest to CM variability in all three directions (AP, ML, and LG). In addition, the joints of the neck and knee showed significant contributions to variability of the CM in ML and LG and AP and LG, respectively. During hand-standing, the variability of the distal joints of the wrist and shoulders played a significant role in predicting the variability of the CM in the AP, ML, and LG direction. In addition, the neck joint motion was significantly correlated to the CM variability in the ML and AP direction. Interestingly, the neck, shoulder, and wrist contributed in more than one plane of motion, as revealed by variability of the neck joint in abduction adduction and the internal and external rotation to variability of the CM_AP_ (Table 2).

### 3.7. Time-Dependent Irregularity (SampEn)

**Figure 3:**
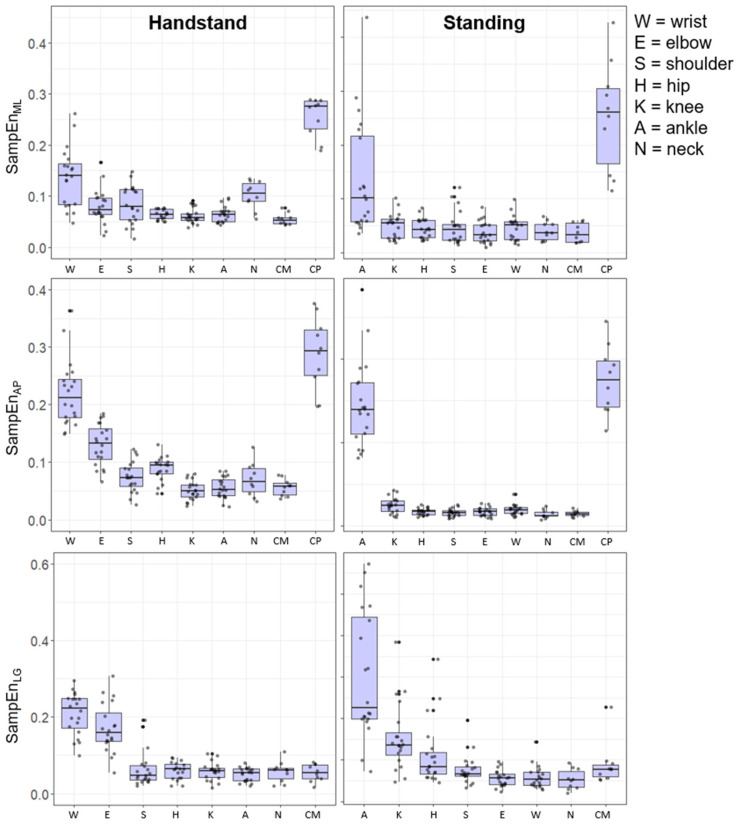
Box plots of the SampEn of the Euclidian joint positions during hand-standing and upright-standing poses for each gymnast on the right and left side.

The time-dependent irregularity of the joint positions is illustrated in Figure 3, where a high level of SampEn was shown for the CP in the AP and ML direction. In the hand-standing and the upright-standing positions there was a higher level of SampEn at the distal joint positions relative to floor contact. The joint position SampEn showed inter-subject variability across all joint positions; however, this was generally greater at the more distal segments, a reverse trend to that of SD.

**Figure 4:**
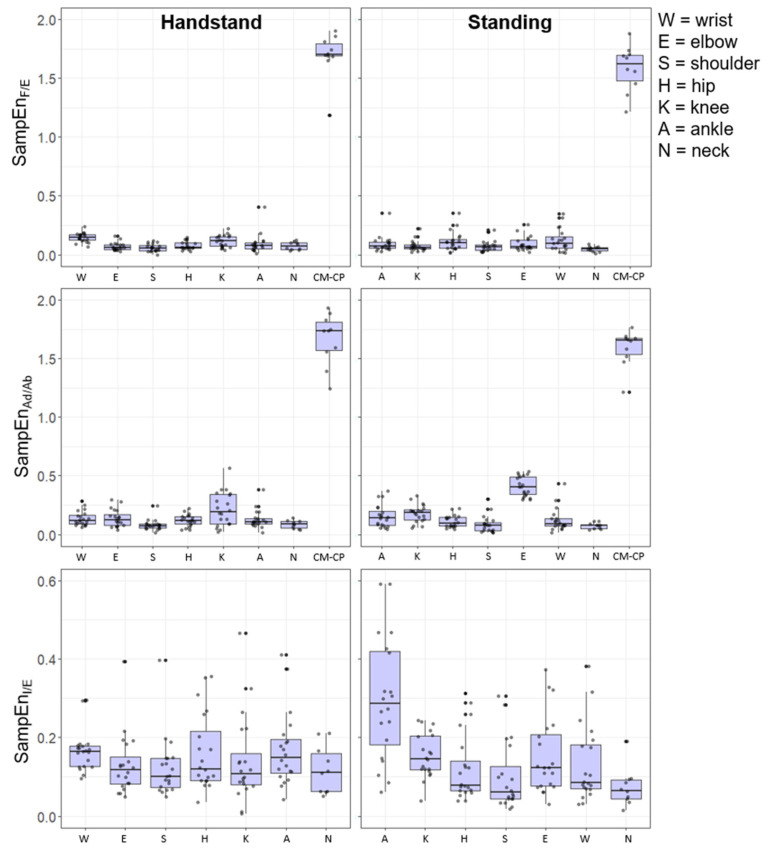
Box plots of the SampEn of the joint angles during hand-standing and upright-standing poses for each gymnast on the right and left side.

The time-dependent irregularity of the joint angles showed a higher SampEn across the joints, and this was magnified in the CP–CM angle ML and AP directions. The joint angles showed a stronger inverse relations to the SD shown in Figure 2. Interestingly, there was a higher SampEn in the distal joints in the flexion/extension direction for hand-standing compared to upright-standing. In the abduction/adduction direction, the upright-standing pose showed a higher SampEn for the distal joints compared to hand-standing (Figure 4).

The findings show an inverse relationship between and time-dependent irregularity (SampEn) and the magnitude of the variability (SD) of the tasks. Bivariate correlation was performed to examine the relationship between time-dependent structure (SampEn) and magnitude (SD) for each pose (see Table 3 and Table 4).

For the upright-standing pose all the correlations between the structure (SampEn) and magnitude (SD) were significant and negative (*p* < 0.05) for the joint angles (CP–CM) and joint positions (CP and CM). Particularly high negative correlations were observed for the CP–CM angle and the CP in upright-standing. In hand-standing, there was a significant negative relationship for the CP–CM and the motion in the AP (CM, CP). However, there was a non-significant relationship between the time-dependent structure (SampEn) and magnitude (SD) for the CP_ML_ and CM_ML_ or CM_LG_.

## 4. Discussion

The patterns of the amount and time-dependent structure of joint motion variability found here in quiet upright- and hand-standing relate to several theoretical and practical issues in the postural control of multi-link biological systems. Our emphasis was on the intra-gymnast variability in maintaining a single trial performance of the respective postures. The single trial approach was used to reflect the typical single trial conditions of competitive performance in gymnastics. The findings revealed several task-driven and general patterns of postural variability that are relevant to understanding the entropy of the postural system.

### 4.1. Hand-Standing and Upright-Standing Postures

The handstand is considered a fundamental gymnastic skill [13] but the amount of intra-gymnast variability was generally greater for all postural indices in the hand-standing than the upright-standing condition [16]. This general task dependent difference in postural variability was present despite the substantial gymnastic experience and skill level of the participants. It is possible that more advanced performers with longer time periods of practice experience and higher levels of skill would narrow to varying degrees this general task difference in angle and position of joint variability and, moreover, change their relation and contribution to CP and CM motions [36].

### 4.2. Joint Angle and Joint 3D Position

The amount of variance of CM explained by multiple regression was overall greater for upright-standing than hand-standing in joint 3D position but the task explained variance was similar for the joint angle data. The greater variation of joint motion in the vertical plane for hand-standing was related to it accommodating a higher percent of variance (R^2^) than that for quiet upright-stance. These findings are consistent with a task specific collective pattern to joint angle and 3D position of joint component variability.

The percent of variance of the multiple regression analyses (R^2^) in predicting the motion of CM in both tasks was on average higher with the 3D position data than the joint angle data. This, as noted, reflects in part the emergent compressed nature of the 3D position variables in contrast to the individual motions of the joint angle degrees of freedom. The high level of percent of variance accommodated by multiple regression analyses is consistent with the findings of previous multivariate analyses of posture. Moreover, the multiple regression findings support postural stance as a multi-link control system [6,7,8,9,10], given the distributed nature of variance accounted for across the joints and the limited contribution of any single joint to the postural control of CM.

The pattern of joint motion variability (angle and 3D position in the Euclidean frame of reference) across the joints was different for the quiet upright- and hand-standing tasks (Figure 1 and Figure 2). This would be expected in a degrees of freedom (DF) view of postural control [7,18] in that the joint angle magnitudes are from a single plane of motion at a single joint whereas the motion of joint position in 3D reflects action at more than one joint. In effect, the joint position data are products of the postural sway of a designated body component that arises from the compression of a set (subset) of joint angle motions [4]. The variability in the motion of CM is the extreme collective example of this compression of the variability of the individual component DFs into a single variable. These different measures of joint motion variability hold different implications for interpreting internal and external frames of reference for mechanisms of postural control. Joint angles are body-relative in an internal frame of reference and these are what are actively controlling posture and CM. In contrast, the joint 3D variability is in the external Euclidean frame of reference and is a local emergent consequence of what is controlled.

### 4.3. Control of CM

The similar absolute and relative low level of CM variability in both hand- and upright-standing tasks is consistent with the longstanding position that the postural system is controlling the motion of CM [4]. Nevertheless, the pattern of joint variability supports the contemporary view of the multivariate control of the CM motion in posture [6,7,8,9,10]. The CM motion in relation to the joint data is consistent with that of a collective variable [20,22] that is regulated by the respective task specific pattern of individual joint motion variability in the two postural tasks.

This joint space organization of postural control (angle and 3D position) holds parallels to the observation of arm joint component DFs controlling within a coordinative structure the motion of the tip of a pistol in a shooting task [37]. It is proposed that the adaptive contribution of the individual joint DFs to the regulation of CM motion depends on the confluence of task, individual, and environmental constraints to postural coordination. In this context, our findings showed a substantial role for neck motion in the overall postural coordinative structure [25], a variable that is rarely analyzed in postural control.

### 4.4. Neck Motion

The motion of the neck has received limited consideration and analysis in postural control except in testing for vestibular contributions to postural control (e.g., [38]). However, the control of head position and visual acuity emerges in part from motion at the neck, in addition to the motion of the individual joints. Visual information has been shown in a variety of experimental protocols to contribute to the control of the posture of quiet upright-standing [39,40] and several head positions relative to the torso have been identified in hand-standing [5]. It follows that different head positions will afford the availability of different layouts of the optic array for visual control of posture [30].

The findings for quiet upright-standing showed a high degree of variability of the neck angle for 3D position that was supported by the pattern of multiple R^2^ values in predicting CM motion. This was particularly the case for postural motion in the AP and ML planes. In contrast, the motion of the neck provided only modest levels of variation in hand-standing that was greater in the vertical plane than it was for upright-standing.

The relatively enhanced motion variation at the neck we conjecture is a product of the search behavior in the mapping of perceptual and motor information for postural control [40]. This is reflected in the proposition of Gibson (1979) that we perceive in order to move, and we move in order to perceive. Thus, there is functional variability to the motion of the joints even in what is generally called the static balance task of upright- or hand-standing.

## 5. Conclusions

The analysis of the individual joint motion variability and its facility to predict motion of the CM supported the longstanding proposition that the motion of CM is what the individual DFs in a coordinative structure are controlling [3,4]. This pattern of the contrasting variability of global and local DFs holds for both the upright- and hand-standing postures. However, the variability of upright-standing is less in terms of amount and time-dependent structure of individual joint variability than the hand-standing task.

The finding of a common task variability pattern to the global (CM, CP) motions, however, is supported by the more unique task driven local (joint) motions. The pattern of the contribution of these variabilities to the control of CP and CM was also influenced by the plane of motion, in which the variability is considered and whether the hands or feet are in contact with the surface of support. Variability of the joint component DFs in the vertical plane was more prevalent in the handstand than the upright stance.

In summary, the patterns of postural variability reveal the task-dependent nature to the joint motions (angle and position), rather than an anatomical pattern of organization. The findings support the proposition that there are both common and task specific properties to the global CM control strategy in the balance of upright- and hand-standing induced by the different active skeletal–muscular organization and the degeneracy revealed in the multiple distributional patterns of the joint angle and position in 3D.

## Figures and Tables

**Table 1:** Multiple regression for joint positions demonstrating % to CM variability in the anterior–posterior (AP), medio-lateral (ML), and vertical (LG) axis.

Joint Positions	Criterion	R^2^	Predictor: Joint/% Contribution/Joint Action			
Handstand	CM_ML_	0.79	Hip 29%_ML_	Shoulder 16%_LG_		
Upright	CM_ML_	0.96	Hip 21%_ML_	Hip 21%_AP_	Neck 17%_ML_	
Handstand	CM_AP_	0.86	Wrist 27%_AP_	Shoulder 14%_LG_	Elbow 13%_LG_	Hip 15%_AP_
Upright	CM_AP_	0.99	Hip 38%_AP_	Knee 15%_AP_	Neck 13%_AP_	Neck 13%_LG_
Handstand	CM_LG_	0.97	Shoulder 22%_LG_	Hip 22%_LG_	Wrist 12%_ML_	
Upright	CM_LG_	0.89	Neck 39%_LG_	Hip 23%_AP_	Knee 13%_AP_	Hip 12%_LG_

**Table 2:** Multiple regression for joint angles demonstrating % to CM variability in the anterior–posterior (AP), medio-lateral (ML), and vertical (LG) axis.

Joint Angles	Criterion	R^2^	Predictor: Joint/% Contribution/Axis					
Handstand	CM_ML_	0.61	Neck 17%_AdAb_	Shoulder 16%_IE_	Shoulder 13%_AdAb_	Wrist 13%_AdAb_		
Upright	CM_ML_	0.62	Ankle 33%_IE_	Neck 25%_FE_	Hip 10%_IE_			
Handstand	CM_AP_	0.70	Shoulder 23%_FE_	Wrist 15%_AdAb_	Elbow 15%_AdAb_	Neck 15%_AdAb_	Neck 10%_IE_	
Upright	CM_AP_	0.76	Hip 21%_IE_	Ankle 21%_IE_	Knee 11%_FE_			
Handstand	CM_LG_	0.92	Shoulder 18%_FE_	Wrist 17%_IE_	Wrist 15%_FE_			
Upright	CM_LG_	0.81	Neck 15%_AdAb_	Knee 13%_FE_	Hip 12%_IE_	Ankle 11%_IE_	Neck 11%_FE_	Neck 10%_IE_

**Table 3:** Bivariate correlation of the CP–CM angle to time-dependent structure in the anterior–posterior (AP) and medio-lateral (ML) axes.

Criterion	Axis	Stance	r
CP–CM	ML	Handstand	−0.58
CP–CM	AP	Handstand	−0.55
CP–CM	ML	Upright	−0.83
CP–CM	AP	Upright	−0.83

**Table 4:** Bivariate correlation of the CP and CM position to time-dependent structure in the anterior posterior (AP), medio-lateral (ML), and vertical (LG) axes.

Criterion	Axis	Stance	r
CP	ML	Handstand	−0.18
CP	AP	Handstand	−0.74
CM	ML	Handstand	−0.38
CM	AP	Handstand	−0.70
CM	LG	Handstand	−0.09
CP	ML	Upright	−0.89
CP	AP	Upright	−0.72
CM	ML	Upright	−0.69
CM	AP	Upright	−0.61
CM	LG	Upright	−0.79

## Data Availability

The data presented in this study are available on request from the corresponding author.
